# The alpha-ketoacid dehydrogenase complexes of *Drosophila melanogaster.*

**DOI:** 10.17912/micropub.biology.001209

**Published:** 2024-04-28

**Authors:** Steven J Marygold

**Affiliations:** 1 FlyBase, Department of Physiology, Development and Neuroscience, University of Cambridge, Cambridge, U.K.

## Abstract

The conserved family of alpha-ketoacid dehydrogenase complexes (AKDHCs) catalyze essential reactions in central metabolism and their dysregulation is implicated in several human diseases. *Drosophila melanogaster* provides an excellent model system to study the genetics and functions of these complexes. However, a systematic account of Drosophila AKDHCs and their composition has been lacking. Here, I identify and classify the genes encoding all Drosophila AKDHC subunits, update their functional annotations and integrate this work into the FlyBase database.

**Figure 1. Components of alpha-ketoacid dehydrogenase complexes in D. melanogaster:**
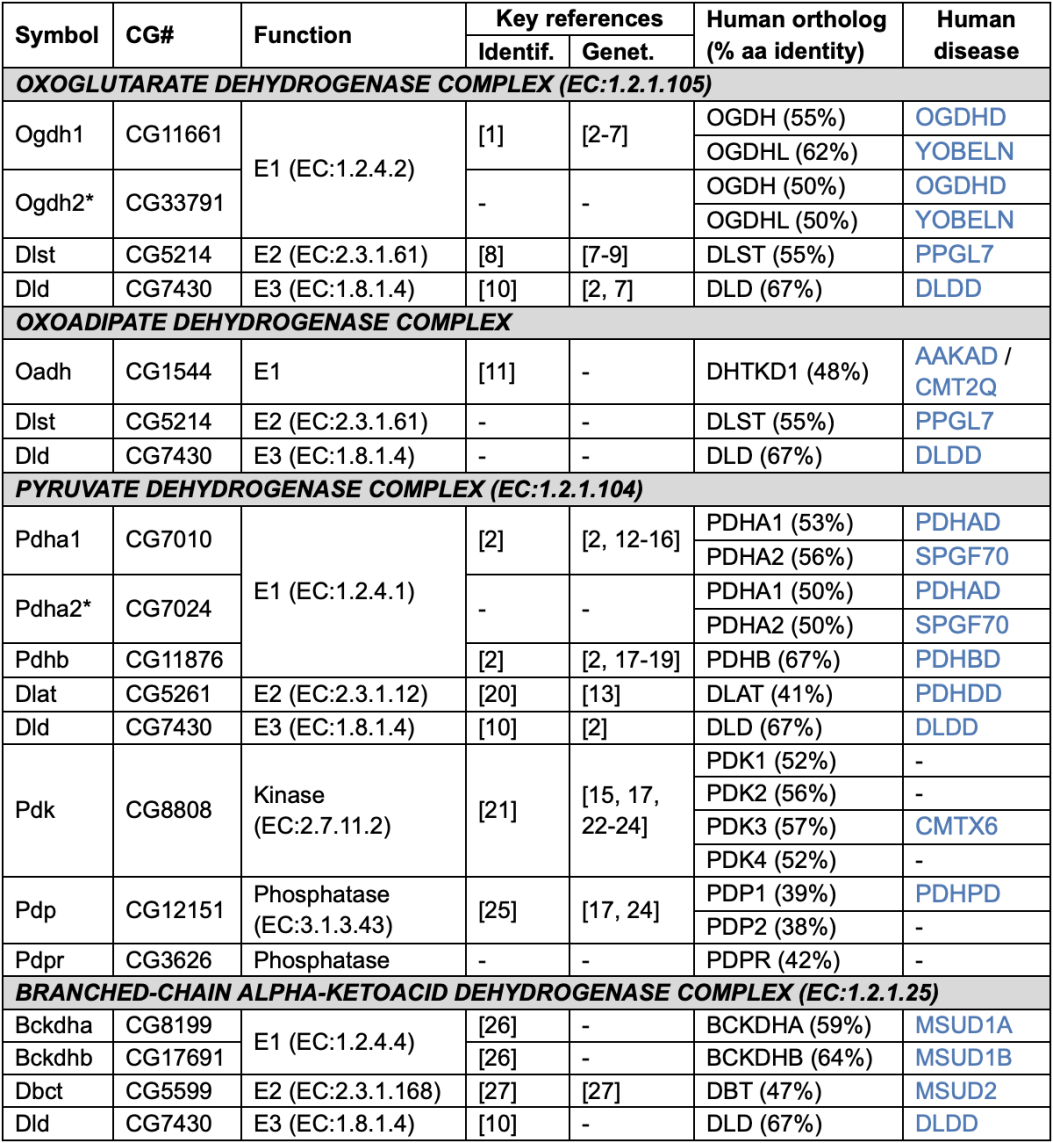
Symbol: gene symbol in FlyBase - asterisk (*) indicates a gene with testis-specific expression. CG#: gene annotation ID in FlyBase. Function: component and associated EC number (where available/applicable). Key reference(s) for initial identification or genetic characterization (in a metabolic context): 1. Gruntenko et al. 1998; 2. Chen et al. 2008; 3. Yoon et al. 2017; 4. Yap et al. 2021a; 5. Yap et al. 2021b; 6. Whittle et al. 2023; 7. González Morales et al. 2023; 8. Homem et al. 2014; 9. Bonnay et al. 2020; 10. Ivanova et al. 2004; 11. Boyko et al. 2020; 12. Liu et al. 2017; 13. Li et al. 2020; 14. Devilliers et al. 2021; 15. Goyal et al. 2022; 16. Huang et al. 2022; 17. Plaçais et al. 2017; 18. Dung et al. 2018; 19. Rabah et al. 2023; 20. Klenz et al. 1995; 21. Katsube et al. 1997; 22. ​​Gándara et al. 2019; 23. Lambrechts et al. 2019; 24. Lee et al. 2022; 25. Chen et al. 2006; 26. Kim et al. 2023; 27. Tsai et al. 2020. Human ortholog: gene symbol at HGNC, with % amino acid identity between the encoded protein and the Drosophila protein. Human disease: OMIM symbol for disease(s) associated with the human gene (Amberger et al. 2019) - see Extended Data File 1 for details.

## Description

Alpha-ketoacid dehydrogenase complexes (AKDHCs) are huge, multi-enzyme complexes that catalyze the oxidative decarboxylation of an alpha-keto acid, such as 2-oxoglutarate (also known as alpha-ketoglutarate), pyruvate or a branched-chain alpha-ketoacid (Patel et al. 1996) . Their structure and function are conserved from bacteria to humans. Structurally, AKDHCs are composed of multiple copies of three enzymes: E1 (an alpha-keto acid dehydrogenase), E2 (a dihydrolipoyl transacylase) and E3 (dihydrolipoamide dehydrogenase). The E2 component forms the central core of each complex, acting as the scaffold for the association of the E1 and E3 subunits. Functionally, an alpha-keto acid is first decarboxylated by the E1 component and the remaining acyl group transferred to a lipoamide cofactor bound to E2. Next, the E2 catalyzes the transfer of the acyl group from lipoamide to coenzyme A (CoA) resulting in acyl-CoA and reduced lipoamide. Finally, the E3 reactivates the lipoamide by oxidation using a bound FAD cofactor and NAD+ as the electron acceptor. While the E1 and (usually) the E2 components are different and provide substrate-specificity to each complex, the same E3 component is shared between them.

In eukaryotes, the AKDHC family comprises four mitochondrially localized complexes. The oxoglutarate dehydrogenase complex (OGDHC) catalyzes the conversion of 2-oxoglutarate into succinyl-CoA in the tricarboxylic acid (TCA) cycle, thereby providing metabolites and reduced electron carriers for oxidative phosphorylation (Nemeria et al. 2021) . The related, albeit less well known, oxoadipate dehydrogenase complex (OADHC) catalyzes the conversion of 2-oxoadipate to glutaryl-CoA in lysine and tryptophan catabolism (Nemeria et al. 2021) . Notably, the OGDHC and OADHC share the same E2 as well as the same E3 component. The pyruvate dehydrogenase complex (PDHC) catalyzes the decarboxylation of pyruvate to produce acetyl-CoA and NADH, thereby linking glycolysis to the TCA cycle and lipogenesis (Stacpoole and McCall 2023) . Lastly, the branched-chain alpha-ketoacid dehydrogenase complex (BCKDHC) catalyzes the conversion of branched-chain 2-oxoacids derived from leucine, isoleucine and valine to branched-chain acyl-CoAs used for ATP generation in the TCA cycle (Harris et al. 2005) . The PDHC and BCKDHC are distinguished from the OGDHC and OADHC by having a heteromeric E1 component and, at least in some species, an integral kinase and phosphatase that regulate their activity (Harris et al. 2005; Stacpoole and McCall 2023) . Apart from the OADHC, the Enzyme Commision (EC) has assigned EC numbers for the reactions catalyzed by individual E1/E2/E3 components as well as the overall reaction catalyzed by each complex (McDonald and Tipton 2023; Table 1). Remarkably, almost all AKDHC subunits are associated with human disease (Table 1, Extended Data File 1).

In *Drosophila melanogaster* (hereafter, Drosophila), many of the genes encoding AKDHC subunits have been identified and several have been genetically characterized through mutation, RNAi-mediated knock-down or over-expression. However, there has been no published account synthesizing information on these complexes and some subunits have remained unidentified. I therefore employed literature searches, orthology predictions, functional annotation data and high-throughput expression datasets to identify and annotate all the subunits of the Drosophila complexes (see Methods). Summarized data are presented in Table 1 and are discussed below in the context of previously published work and with comparison to the human complexes.

**OGDHC:** The E1 component of the OGDHC is encoded by two paralogous genes in Drosophila, as in humans. Drosophila *Ogdh1* (previously *Ogdh* ) is expressed throughout all developmental stages and in all tissues, whereas *Ogdh2* expression is testis-specific (Öztürk-Çolak et al. 2024), suggesting that a specialized version of the OGDHC exists in the Drosophila testis. Similarly, human *OGDH* is expressed ubiquitously whereas expression of *OGDHL* is restricted to the brain (Yap et al. 2021) . Following the identification of Drosophila *Ogdh1* (Gruntenko et al. 1998) , most of the studies characterizing it have focused on the neurodegenerative phenotypes of mutants that serve as a model for *OGDH- or OGDHL* -associated human diseases (Yoon et al. 2017, Yap et al. 2020, Yap et al. 2021, Whittle et al. 2023) . Additionally, the Ogdh1 protein localizes to Z-discs where OGDHC activity is required for myofibril formation (González Morales et al. 2023). In contrast, the testis-specific *Ogdh2* gene is identified here for the first time. The E2 component of the Drosophila OGDHC is encoded by a single, ubiquitously expressed gene, *Dlst* (Homem et al. 2014) . It plays a particularly important role in neuroblast growth and proliferation (Homem et al. 2014, Bonnay et al. 2020) .

**OADHC:** The OADHC differs from the OGDHC by only its E1 component, which is encoded by *Oadh* in Drosophila (Boyko et al. 2020) . Neither the *Oadh* gene nor the OADHC has been characterized in Drosophila to date.

**PDHC:** In Drosophila, the alpha subunit of the heteromeric E1 component of the PDHC is encoded by two paralogous genes: *Pdha1* (previously *Pdha* ) is expressed ubiquitously, whereas *Pdha2* is expressed almost exclusively in the testis (Öztürk-Çolak et al. 2024). This exactly mirrors the situation in humans (Stacpoole and McCall, 2023) and indicates that the Drosophila PDHC, like the OGDHC, exists as a testis-specific variant. The beta subunit of the PDHC is, in contrast, encoded by a single gene in both organisms. *Pdha1* and *Pdhb* are among the best characterized AKDHC genes in Drosophila: in particular, mutation/knockdown of these genes have been used to assess PDHC function in neurons and glia, and to generate fly models of PDHC-associated neuropathic and neurodegenerative diseases (Liu et al. 2017; Plaçais et al. 2017; Dung et al. 2018; Li et al. 2020; Rabah et al. 2023). In contrast, the testis-specific *Pdha2* gene has not been previously identified. The E2 component of the Drosophila PDHC, encoded by the *Dlat* gene, was first identified as a behavioral mutant (Phillis et al. 1993; Klenz et al. 1995) but has received scant attention since.

The single subunit PDHC kinase, as well as the catalytic and regulatory subunits of the PDHC phosphatase, are encoded by single genes in Drosophila. This contrasts with humans, where multiple genes encode the PDHC kinase and phosphatase catalytic subunit to generate tissue-specific isoforms (Stacpoole and McCall, 2023) . Drosophila *Pdk* (Katsube et al. 1997) and *Pdp* (Chen et al. 2006) have been identified previously, and manipulation of these two genes has been used to modulate Drosophila PDHC function *in vivo* (Plaçais et al. 2017; Gándara et al. 2019; Lambrechts et al. 2019; ​​Goyal et al. 2022; Lee et al. 2022). In contrast, the Drosophila *Pdpr* gene encoding the regulatory subunit of the phosphatase is uncharacterized and identified here for the first time. Of note, the human PDHC includes an additional subunit, the E3-binding protein called E3BP/PDHX (Stacpoole and McCall, 2023) , but this lacks a clear ortholog in Drosophila.

**BCKDHC:** The genes encoding the subunits of the heterodimeric E1 component of the Drosophila BCKDHC, *Bckdha* , and *Bckdhb* , have been identified but not further characterized (Kim et al. 2023) . The BCKDHC E2-encoding gene, *Dbct* , has been used to generate a fly model of maple syrup urine disease (Tsai et al. 2020) . Notably, Drosophila appears to lack orthologs of the BCKDHC-specific kinase (BCKDK) and phosphatase (PPM1K) that regulate the activity of the human complex (White et al. 2018) . Whether the Drosophila BCKDHC is regulated by an analogous mechanism remains to be explored.

**E3 component** : The *Dld* gene has been identified as encoding the shared E3 component of the OGDHC, PDHC and BCKDHC (Ivanova et al. 2004) . Genetic characterization of this component has been attempted (Chen et al. 2008; González Morales et al. 2023), though interpretation of the resulting phenotypes is complicated by the fact *Dld* disruption affects the function of all AKDHCs.

As part of this work the Gene Ontology (GO) terms (Gene Ontology consortium 2023) describing the four distinct AKDHCs and the catalytic activities of their individual components were reviewed and rationalized (see Extended Data File 2). The GO annotations of all Drosophila AKDHC subunits have been updated to reflect these revisions, and these improvements are now available via FlyBase and other resources displaying Drosophila GO annotation data. Furthermore, the Drosophila AKDHCs have been curated as ‘Gene Groups' within FlyBase (Attrill et al. 2016) , thereby facilitating access to data about each complex ( http://flybase.org/reports/FBgg0001924.htm ).

In summary, this work presents the first data synthesis and overview of the AKDHCs of Drosophila. It highlights several important themes, including the sharing of components between complexes, the presence of testis-specific variants, and the potential for creating additional models of AKDHC-related disease in flies. This report, and the associated improvements to GO annotations, data and resources in FlyBase, will assist further genetic, biochemical and biomedical research on AKDHC biology in Drosophila and its wider application within biomedicine.

## Methods

Subunits of the Drosophila AKDHCs were identified by querying the FlyBase database (http://flybase.org), release FB2023_01, using (i) relevant GO terms (see Extended Data File 2); (ii) human orthologs; and (iii) literature searches, mainly by using the appropriate interface of the QuickSearch tool (Marygold and FlyBase consortium, 2023) . Orthology searches relied on the underlying DIOPT orthology tool (Hu et al. 2011) , which is also the source of the amino acid identity reported in Table 1. The results of these searches were cross-checked and verified manually, with edits made to FlyBase gene records and GO annotations as necessary. Key references relating to each gene reported in Table 1 were initially identified using the ‘Representative Publications' subsection of FlyBase reference reports (Thurmond et al. 2019) followed by manual verification. Testis-enriched AKDHC subunits were identified by inspecting the high-throughput expression data section of FlyBase gene reports. EC numbers are derived from the cross-references present on the associated GO terms. Human gene-to-disease association data was obtained from https://www.omim.org/ (accessed 16th April 2024; Amberger et al. 2019).

## Extended Data

Description: Current GO terms relevant to the complexes and catalytic activities shown in Table 1, following their revision. Entries are taken from the go-basic.obo file (data-version: releases/2024-03-28) obtained from http://current.geneontology.org/ontology/go-basic.obo.. Resource Type: Text. DOI: 10.22002/9ampc-7df66

Description: OMIM phenotype-gene relationships for genes encoding subunits of the human AKDHCs.. Resource Type: Text. DOI: 10.22002/vpate-7c171
